# Enhanced Detection of Type 2 Diabetes Mellitus in an At-Risk Prediabetic Population Using One-Hour Oral Glucose Tolerance Test (OGTT): A Comparison With HbA1C and Two-Hour OGTT

**DOI:** 10.7759/cureus.100330

**Published:** 2025-12-29

**Authors:** Senthilvelan Thenmozhi, Subramanian Girija

**Affiliations:** 1 General Medicine, Employees State Insurance Corporation (ESIC) Medical College and Post Graduate Institute of Medical Sciences and Research (PGIMSR), Chennai, IND; 2 Internal Medicine, Sri Manakula Vinayagar Medical College, Puducherry, IND

**Keywords:** diabetes mellitus, glycated hemoglobin, one-hour ogtt, oral glucose tolerance test, prediabetes

## Abstract

Introduction

The oral glucose tolerance test (OGTT), though valuable, is underutilized in outpatient settings for the diagnosis of type 2 diabetes mellitus (T2DM). Our study aimed to confirm prediabetes and assess the risk of developing T2DM in individuals with elevated risk, comparing the diagnostic utility of OGTT with fasting plasma glucose (FPG), post-prandial plasma glucose (PPPG), and HbA1C.

Methods

A hospital-based cross-sectional study was conducted in the Department of General Medicine of a tertiary care hospital in South India, including adults over 18 years with elevated random plasma glucose (RPG) (≥140 mg/dl), FPG (110-125 mg/dl), and HbA1C (5.7-6.4%). After an overnight fast, participants received 75 g of glucose, and venous blood samples were collected at fasting, one hour, and two hours to measure plasma glucose.

Results

Of the 113 participants, 55.8% (63) were males and 57.5% (65) were overweight or obese (BMI ≥ 23 kg/m²). FPG classified 44.3% (50) as prediabetic and 9.7% (11) as diabetic. While HbA1C categorized 63.7% (72) as prediabetic and 18.6% (21) as diabetic (cutoff >200 mg/dl), the one-hour OGTT categorized 32.7% (37) as prediabetic (cutoff: 155-199 mg/dl) and 44.2% (50) as diabetic. The one-hour OGTT showed greater sensitivity for detecting at-risk individuals.

Conclusions

The one-hour OGTT, compared to FPG, PPPG, and HbA1C, offers enhanced sensitivity in confirming prediabetes and unmasking T2DM in the prediabetic population. Incorporating the one-hour OGTT into routine screening may improve early diagnosis and intervention, especially in at-risk populations.

## Introduction

Diabetes mellitus (DM) is a commonly prevalent disease in the outpatient setting. The diagnostic criteria for DM (as per American Diabetes Association) include fasting plasma glucose (FPG) ≥126 mg/dl, glycated hemoglobin (HbA1C) >6.5%, or random plasma glucose (RPG) ≥200 mg/dl in symptomatic individuals [1]. Oral glucose tolerance test (OGTT) with 75 g of glucose (in 300 ml of water) can identify glucose intolerance and reflect the impairment of insulin release. However, it is not routinely used in clinical practice for the diagnosis of DM. This probably could be because it is cumbersome, requiring multiple samples and repeated venous blood sampling. Prospective studies in populations undergoing OGTT have demonstrated that such abnormalities are not only common but also associated with a twofold increase in mortality risk [2]. Impaired fasting glucose (IFG) and impaired glucose tolerance (IGT) represent key prediabetic states that significantly raise the likelihood of future dysglycemia. Notably, OGTT has been shown to diagnose up to 30% more individuals with dysglycemia compared to reliance on HbA1C alone [3].

Early diagnosis of prediabetes is critical to mitigating the growing global burden of DM. Current estimates claim that 9.9% of the global population will have DM by 2045 [4], while the prevalence in the Indian population is expected to rise to 10.8% [5]. Similarly, the global prevalence of IGT is predicted to increase from 7.3% to 8.3% in 2045, while IGT rates in India are projected to climb to 5.8% from 5.4% over the same period [4,5].

This study aims to evaluate the utility of OGTT as a diagnostic tool in a high-risk prediabetic population for early identification of type 2 DM (T2DM). It also compares OGTT results with FPG, post-prandial plasma glucose (PPPG), and HbA1C to classify individuals into T2DM or prediabetes categories.

## Materials and methods

Study design

This hospital-based cross-sectional study was conducted over a period of 18 months in the Department of General Medicine at a tertiary care hospital in South India. Institutional Ethics Committee approval was obtained. Consecutive consenting patients (from the General Medicine Outpatient Clinic and Master Health Check-up Clinics), meeting the inclusion criteria and providing written informed consent, were enrolled in the study.

Inclusion criteria

Individuals aged >18 years and individuals presenting with classical symptoms of DM (polyuria, polydipsia, polyphagia) but having normal random plasma glucose (RPG: 79-140 mg/dL), or elevated RPG (140-199 mg/dL), or impaired FPG (FPG: 110-125 mg/dL), or intermediate HbA1C levels between 5.7% and 6.4% were included for the study.

Exclusion criteria

Patients with a prior diagnosis of DM, gestational DM, or on antidiabetic medications; admitted to the emergency department with acute complications of DM; and those with anemia or hemoglobinopathies were excluded.

Data collection

Data collection was done using a standard proforma. Demographic details, brief clinical history, and anthropometric measurements were recorded. Patients were instructed to maintain the usual diet and exercise patterns up to the evening before the test. They were to consume a minimum of 150 grams of carbohydrate daily over the three days prior to the test. They were requested to come after an eight-hour overnight fast to perform the OGTT.

Brief procedure

After an eight-hour fast, 2 mL of venous blood was collected for baseline FPG estimation. Participants then ingested a 75 g anhydrous glucose solution dissolved in 300 ml water (osmolarity 1.39 mol/L) within 5-15 minutes. Venous blood samples were obtained again at 60 and 120 minutes post-administration. Smoking, physical exertion, and consumption of beverages/additional food were not permitted for the duration of the test. All procedures were supervised by the principal investigator, and samples were analyzed immediately. All patients also underwent HbA1C testing. Blood glucose was measured by the enzymatic (hexokinase) method, and HbA1C was measured by high-performance liquid chromatography (HPLC).

Statistical methods

Data collected using the standard proforma were tabulated in MS Excel (Microsoft Corporation, Redmond, Washington, United States), and statistical analysis was done using IBM SPSS Statistics for Windows, Version 26 (Released 2018; IBM Corp., Armonk, New York, United States). Continuous variables were expressed as frequency and mean with standard deviation, while discrete variables were expressed as proportions and percentages. Student t-test (two-tailed, independent) was used for continuous variables based on the normality (Shapiro-Wilk test) of the distribution. Chi-square or Fisher's exact test was used to evaluate parameters on a categorical scale. Pearson or Spearman correlation was applied based on the nature of the distribution of the variables. Receiver operating characteristic curve analysis was performed to assess the cutoff value of the one-hour post-glucose-load plasma glucose (PGLPG) to diagnose DM. Sample size calculated as 113 using nMaster version 2.0 involved a relative precision value of 20% based on previous studies. A p-value of <0.05 was considered statistically significant. 

## Results

A total of 113 patients were included in the final analysis.

Demographic data

The mean (± SD) age of the cohort was 47.96 ± 12.62 years (range: 20-76 years), with the majority (83, 73.5%) of the patients falling in the 31-60 years age group. The population exhibited an approximately normal age distribution on histogram analysis. Men constituted the majority (63/113; 55.8%).

Body mass index and waist-hip ratio (WHR)

The mean height (± SD) of the entire patient population was 160 (± 9.3) cm. The mean weight (± SD) was 66.55 (± 12.85) kg. The mean (± SD) body mass index (BMI) of the cohort was 25.8 ± 4.23 kg/m2, with 57.5% (65/113) classified as overweight/obese (BMI: ≥23 kg/m2) and 39.8% (45/113) in the normal range (18.5-22.9 kg/m2). The mean (± SD) WHR was 0.94 ± 0.02. Women had a significantly higher WHR (p < 0.001).

Clinical presentation

Fatigue (36; 31.9%), angina (21; 18.6%), and stroke (10; 8.8%) were the most common among the clinical presentations. There was a family history of DM in 10 (8.8%) patients. Smoking was observed in 29.2% (33) and alcohol use in 31.9% (36) patients. Notable comorbidities included hypertension (4.4%, 5/113), dyslipidemia (4.4%; 5/113), and anemia (clinical pallor-7/113; 6.2%). Acanthosis nigricans was observed in three (2.7%) patients (Table 1).

**Table 1:** Gender-wise distribution of clinical presentation p-value (Chi-square test)-0.107; n = number of patients

Clinical presentation	Male n (%) n = 63	Female n (%) n = 50	Total N (%) (N = 113)
Fatigue	17 (27)	19 (38)	36 (31.9)
Angina	15 (23.8)	6 (12)	21 (18.6)
Stroke	7 (11.1)	3 (6)	10 (8.8)
Dyspnea on exertion	3 (4.8)	5 (10)	8 (7.1)
Acid peptic disease	3 (4.8)	4 (8)	7 (6.2)
Preoperative workup	7 (11.1)	0	7 (6.2)
Headache	1 (1.6)	4 (8)	5 (4.4)
Giddiness	2 (3.2)	2 (4)	4 (3.5)
Palpitations	2 (3.2)	1 (2)	3 (2.7)
Blurring of vision	1 (1.6)	2 (4)	3 (2.7)
Seizures	2 (3.2)	1 (2)	3 (2.7)
Neuropathic pain	0	2 (4)	2 (1.8)
Dysuria	2 (3.2)	0	2 (1.8)
Infertility	0	1 (2)	1 (0.9)
Knee pain	1 (1.6)	0	1 (0.9)

Macro and microvascular complications

**Figure 1:**
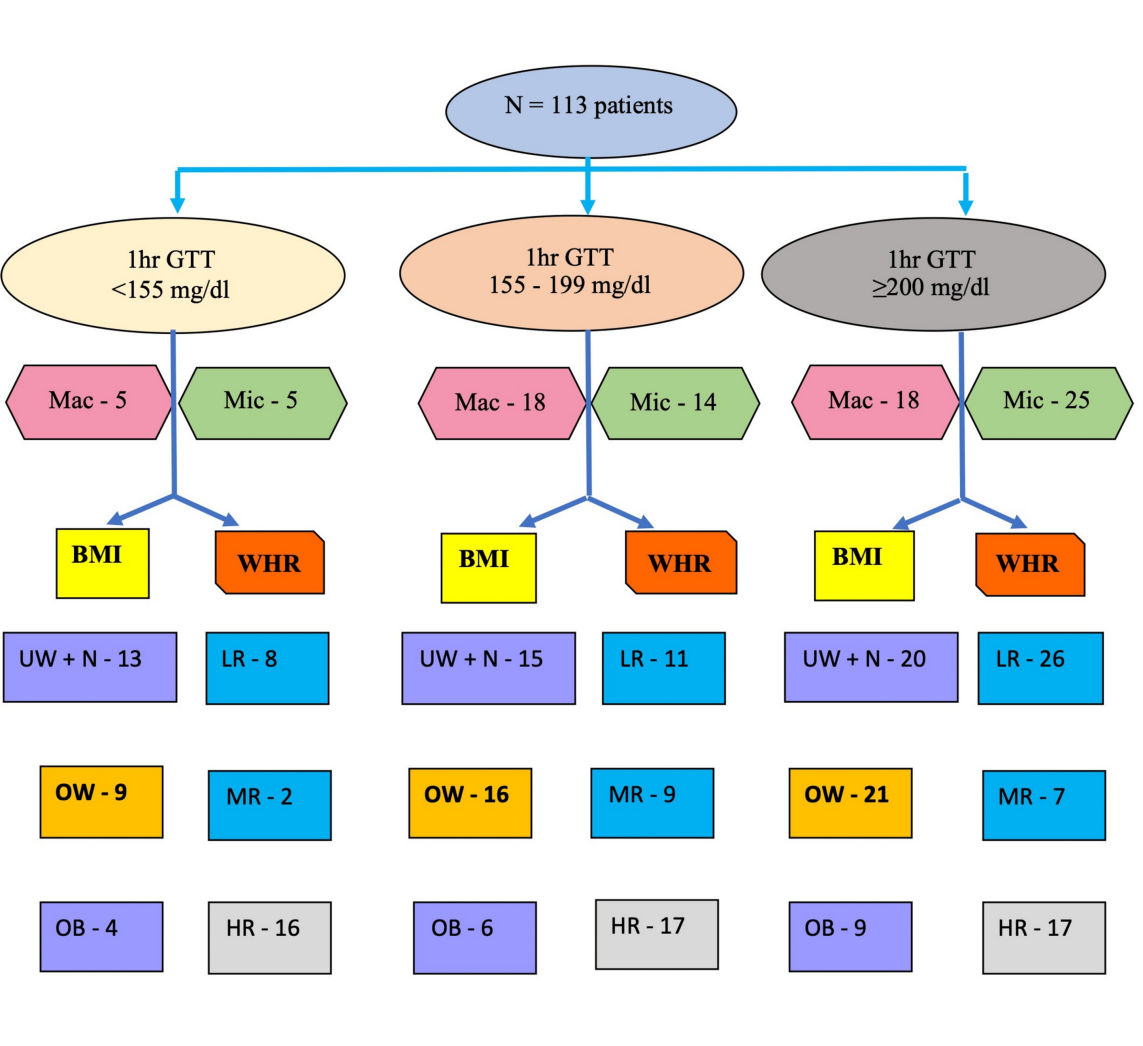
Patients with complications at presentation classified based on body mass index and waist-hip ratio N: total number of patients; 1hr GTT: OGTT-one-hour PGLPG value; Mac: macrovascular complications; Mic: microvascular complications; BMI: body mass index; UW + N: underweight and normal category BMI; OW: overweight category BMI; OB: obese category BMI; WHR: waist-hip ratio; LR: low risk WHR category; MR: moderate risk WHR category; HR: high risk WHR category

Among 113 patients, 38.9% (44) exhibited macrovascular complications (stroke, angina, dyspnea on exertion and headache) and 48.7% (55) presented with associated symptoms (fatigue, acid peptic disease, palpitations, dysuria, neuropathic pain, giddiness and infertility) at the time of presentation. Gender-stratified clinical presentations are detailed in Table 1. Figure 1 illustrates the prevalence of complications stratified by BMI/WHR.

Glycemic profiling

Baseline Glycemic Values

Patient recruitment was based on initial HbA1C with FPG or RPG values, inferred as prediabetes.

OGTT vs. HbA1C Diagnostic Performance

Using OGTT-one-hour post-glucose load plasma glucose (one-hour PGLPG) cutoffs (<155 mg/dL: normal; 155-199 mg/dL: prediabetes; ≥200 mg/dL: diabetes), 44.2% (50/113) were classified as diabetic, 32.7% (37/113) as prediabetic, and 23% (26/113) as normoglycemic (Table 2).

**Table 2:** Classification of patient population based on OGTT and HbA1C OGTT: oral glucose tolerance test; HbA1C: glycated hemoglobin; FPG: fasting plasma glucose; one-hour PGLPG: 1st hour post-prandial plasma glucose; two-hour PGLPG: 2nd hour post-prandial plasma glucose; n: number of patients; p-value-not applicable Based on cutoff values, <155mg/dl: normal; 155 to <200: prediabetic; >200, diabetic

Category	OGTT-FPG n (%)	OGTT-one-hour PGLPG* n (%)	OGTT-two-hour PGLPG n (%)	HbA_1_C n (%)
Normal	52 (46)	26 (23)	48 (42.5)	20 (17.7)
Prediabetes	50 (44.3)	37 (32.7)	40 (35.4)	72 (63.7)
Diabetes mellitus	11 (9.7)	50 (44.2)	25 (22.1)	21 (18.6)

Key findings

**Figure 2:**
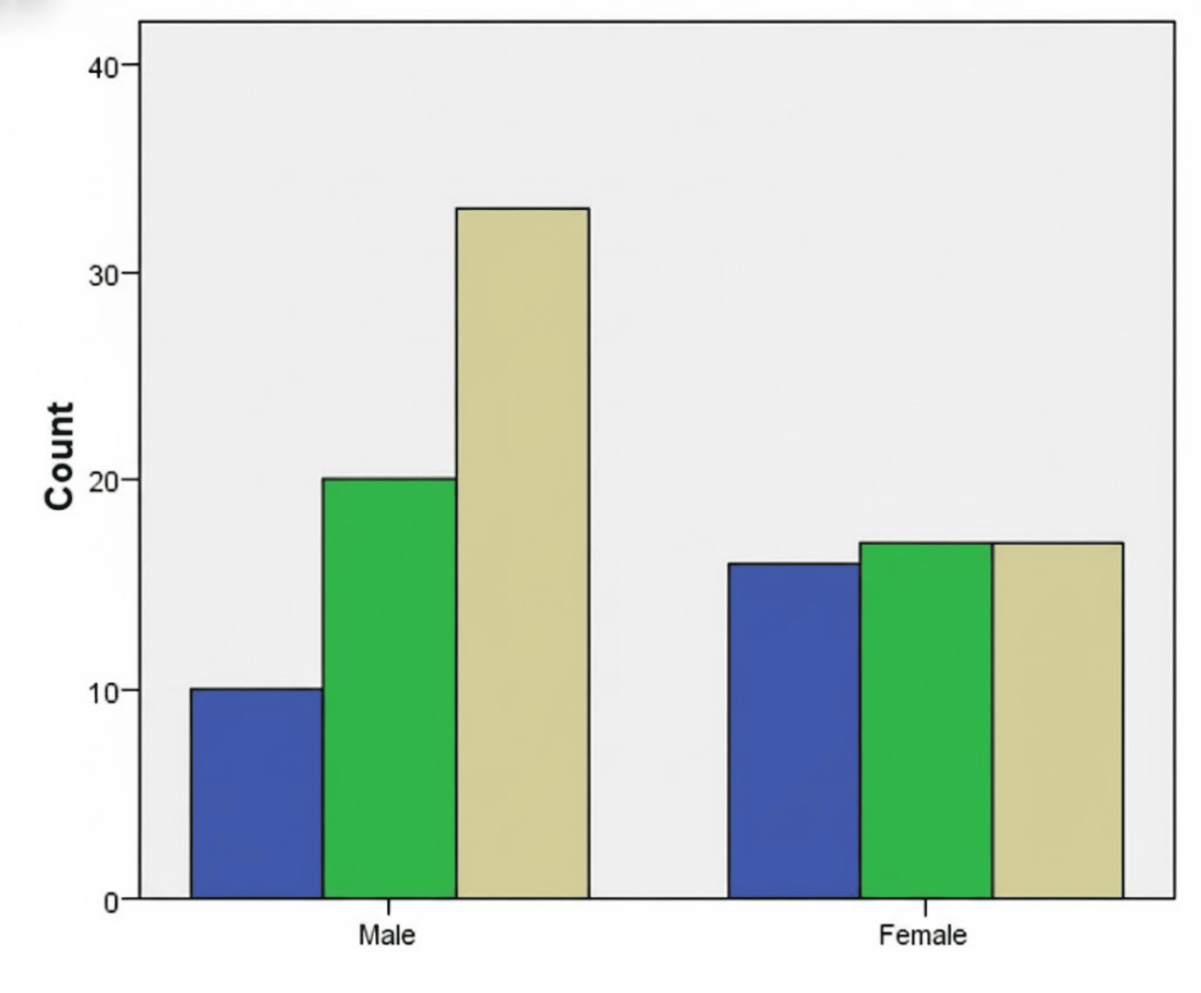
Bar chart showing distribution of men and women in normal, prediabetes and diabetes mellitus categories based on OGTT-one-hour PGLPG PGLPG: post-glucose-load plasma glucose; count: number of patients in each category; blue: normoglycemic; green: prediabetic; yellow: diabetes mellitus

OGTT-one-hour PGLPG identified twice as many diabetes cases as OGTT-two-hour PGLPG and 2.3-fold more than HbA1C. About 39/50 (78%) of OGTT-one-hour-diagnosed diabetic patients were misclassified as prediabetic/normal by HbA1C (Table 3). Around 17/26 (65.4%) patients with normal one-hour PGLPG were labelled prediabetic by HbA1C, suggesting preserved β-cell function amenable to lifestyle interventions. OGTT-two-hour PGLPG detected five additional prediabetic cases missed by HbA1C alone. The OGTT-one-hour values identified substantially more cases of diabetes than HbA1C or OGTT-two-hour values. Gender-stratified analysis revealed significant disparities; women showed balanced distribution across glycemic categories, whereas men skewed toward T2DM (p = 0.023) (Figure 2).

**Table 3:** Reclassification of patients based on OGTT-one-hour PGLPG values in comparison with fasting plasma glucose, OGTT-two-hour PGLPG and HbA1C PGLPG: post-glucose-load plasma glucose; OGTT: oral glucose tolerance test; HbA1C: glycated hemoglobin; FPG: fasting plasma glucose; two-hour PGLPG: 2nd hour post-prandial plasma glucose; n: number of patients in each group p-value (Chi-square test) = 0.023

OGTT-one hour	Glycemic status	OGTT–FPG n	OGTT-two-hour PGLPG n	HbA_1_C n (N = 113)
Normal n = 26	Normal	17	25	7
	Prediabetes	8	1	17
	Diabetes mellitus	1	0	2
Prediabetes n = 37	Normal	21	18	9
	Prediabetes	15	19	20
	Diabetes mellitus	1	0	8
Diabetes mellitus n = 50	Normal	14	5	4
	Prediabetes	27	20	35
	Diabetes mellitus	9	25	11

Based on the receiver operating characteristic (ROC) curve analysis for OGTT-one-hour PGLPG in diagnosing DM, the area under the curve (AUC) was 0.615, with a sensitivity of 62.1% and specificity of 61.8% for a cutoff value of OGTT-one-hour PGLPG of ≥200 mg/dl.

Diagnostic Overlap

**Figure 3:**
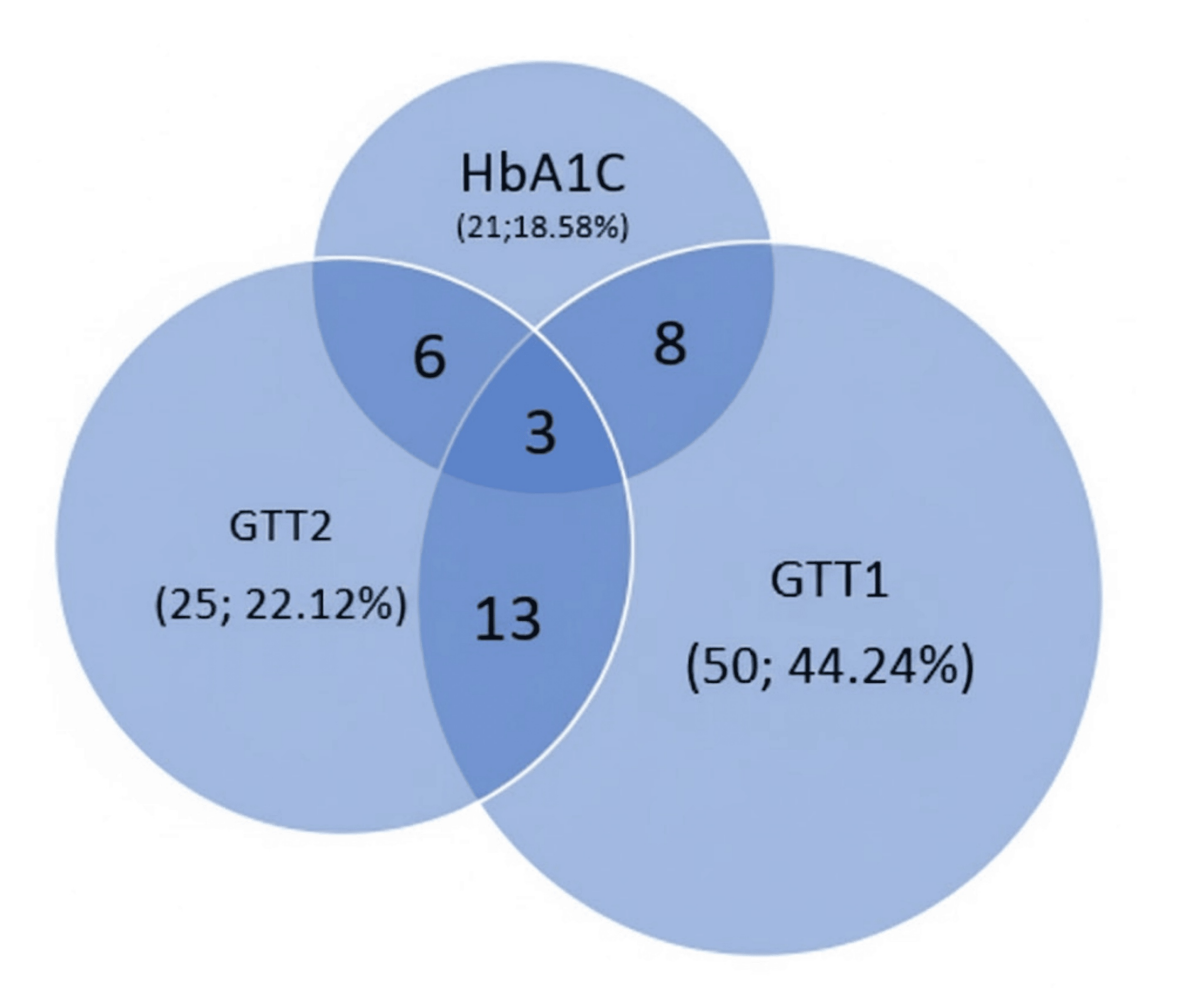
Venn diagram to show overlap of diagnoses based on HbA1C, OGTT-one-hour and two-hour PGLPG values PGLPG: post-glucose-load plasma glucose; HbA1C: glycated hemoglobin; GTT1: 1st-hour PGLPG; GTT2: 2nd-hour PGLPG

The Venn diagram (Figure 3) illustrates discordance among HbA1C, OGTT-one-hour, and OGTT-two-hour PGLPG criteria. Notably, one-hour PGLPG captured unique diabetic/prediabetic cases undetected by other methods. It was observed that more patients got classified as prediabetes and T2DM based on OGTT-one-hour values at a cutoff of 155-199/>200 mg/dl (Table 3).

## Discussion

This study emphasizes the critical role offered by the OGTT-one-hour PGLPG during OGTT in unmasking dysglycemia, particularly in high-risk populations. These findings align with the mounting evidence that prediabetes is far from being benign. OGTT offers a crucial window for early intervention [6-9]. High values of OGTT-one-hour PGLPG indicate beta cell dysfunction in an otherwise normally regarded patient on two-hour PPPG or OGTT-two-hour PGLPG. OGTT provides an opportunity to directly evaluate the responsiveness of pancreatic β-cells to an acute glycemic stress. Rapid normalization of glucose levels after OGTT correlates with preserved β-cell function and reduced risk of T2DM over eight years [10].

OGTT-one-hour PGLPG as a predictor of diabetes risk

Longitudinal data depict one-hour PGLPG ≥155 mg/dL to be a robust predictor of incident T2DM within the next five years, and one-hour PGLPG values ≥200 mg/dL at any time point in OGTT confirm overt T2DM. This threshold picks up a larger at-risk cohort than traditional impaired glucose tolerance (IGT) criteria, thereby giving a chance to administer targeted preventive strategies [9-12]. A cutoff value of 155 mg/dl was validated by Priya et al. based on its sensitivity and specificity, while Abdul-Ghani et al. demonstrated the effectiveness of one-hour PGLPG values over fasting plasma glucose (FPG) and two-hour PPPG in predicting T2DM [13,14]. Combining one-hour PGLPG with metabolic syndrome criteria (NCEP/ATP III) can further stratify at-risk individuals [10]. The cutoff of one-hour PGLPG ≥155 mg/dL predicting five-year diabetes risk even in normoglycemic individuals, authenticating its utility in early screening, is further substantiated by the CATAMERI, Botnia, and Malmö Preventive Project studies [13,15].

Risk based on demographic and anthropometric data

The mean BMI (25.8 kg/m²) in our cohort depicted the high chances of Asian populations for T2DM [14]. High WHRs (≥0.85) in women in our cohort emphasized the relationship of central adiposity and consequent dysglycemia in India.

Diagnostic discordance: OGTT vs. standard lab parameters

OGTT-one-h PGLPG reclassified 44% (50/113) of participants as T2DM, which meant that a diagnosis of T2DM was missed by HbA1C in 11/50 (22%) and FPG in 9/50 (18%). Also, 39/50 (78%) of these T2DM patients were misclassified as prediabetic/normal by HbA1C, which was similar to the cohort of Thewjitcharoen et al., and concluded that the diagnosis of T2DM was doubled compared to that by HbA1C performed concurrently. The OGTT-two-hour PGLPG values detected 50% of cases, indicating the greater diagnostic sensitivity of OGTT-one-hour PGLPG [16].

Clinical implications of undiagnosed dysglycemia

It was identified that at least 25/50 (50%) of T2DM detected by OGTT-one-hour PGLPG values had macrovascular complications (stroke, angina, etc.), and these were not detected by HbA1C or FPG. This finding aligns with the evidence that macrovascular damage begins during prediabetes itself and illustrates the potential clinical impact of delayed detection when only traditional labs such as FPG and HbA1C are employed. The prevalence of microvascular complications (neuropathy, etc.) at presentation further reinforced the role of OGTT and one-hour PGLPG values in alleviating preventable morbidity in at-risk populations [17]. This also helps in understanding the impact of unmeasured duration of hyperglycemia.

Comparing HbA1C and OGTT-one-hour PGLPG

HbA1C offers logistical advantages such as performing at any time and a single blood test [18]. Though OGTT one-hour PGLPG is less convenient to perform when compared to HbA1C, it provides a crucial window to detect the population at-risk for dysglycemia, earlier than that by HbA1C [18]. This has already been highlighted in previous studies, especially in Asian Indians, that even “normal” HbA1C (6.1-6.4%) warrants confirmation with OGTT and proposals to reduce further screening thresholds to HbA1C as low as 6.03% for early detection [19,20]. Paucity of data, pertaining to region and ethnicity, could be reasons why the OGTT-one-hour PGLPG is not yet widely used in clinical practice. European guidelines mandate performing OGTT for patients with cardiovascular disease, recognizing its prognostic value for T2DM, chronic kidney disease, and metabolic dysfunction-associated fatty liver disease [21,22].

Double advantage of early OGTT-one-hour PGLPG

Early OGTT-one-hour PGLPG offered timely intervention due to an early diagnosis of prediabetes and offered time for lifestyle and drug interventions to delay the onset of overt T2DM and thereby bring down the incidence of complications [23]. Also, patients with preserved pancreatic β-cell function (as suggested by normal one-hour PGLPG but elevated HbA1C) appear to be amenable to maximal benefit from lifestyle changes and the potential to avert the onset of T2DM indefinitely [24]. OGTT-one-hour PGLPG can be viewed as a surrogate for insulin sensitivity, especially in populations at risk, and patients can be subjected to this specific testing based on OGTT values [25].

Rigorous evaluation of OGTT-one-hour PGLPG as a superior diagnostic tool for unmasking dysglycemia in at-risk populations, to identify 44% of T2DM cases missed by HbA1C and FPG, is one of the important strengths of this study. Lack of longitudinal follow-up to confirm progression to T2DM in this single-center trial with limited sample size could be a potential drawback and would call for multicenter data with longer follow-up duration.

## Conclusions

This study underscores the importance of OGTT-one-hour PGLPG (as a pivotal biomarker to identify T2DM). In order to address the ever-increasing global burden of T2DM and initiate lifestyle interventions early, OGTT-one-hour PGLPG testing may be integrated as a standard screening protocol, especially for at-risk populations in diverse ethnic cohorts, and requires multicenter research for wider clinical application.
